# Bilateral limbal stem cell deficiency with xeroderma pigmentosum in a young Asian child

**DOI:** 10.1002/ccr3.7746

**Published:** 2023-07-30

**Authors:** Bharat Gurnani, Kirandeep Kaur

**Affiliations:** ^1^ Cataract, Cornea, External Disease, Trauma, Ocular Surface and Refractive Services Sadguru Netra Chikitsalya, Shri Sadguru Seva Sangh Trust Chitrakoot Madhya Pradesh India; ^2^ Cataract, Paediatric Ophthalmology and Strabismus Services, Children Eye Care Centre Sadguru Netra Chikitsalya, Shri Sadguru Seva Sangh Trust Chitrakoot Madhya Pradesh India

**Keywords:** calcific deposits, posterior capsular opacification, pseudophakos, sunflower pattern

## Abstract

**Key Clinical Message:**

Xeroderma pigmentosum is an autosomal recessive disorder with various ocular manifestations of which bilateral limbal stem cell deficiency is a rare manifestation. Timely diagnosis and meticulous management are vital in these cases to prevent irreversible ocular sequelae.

**Abstract:**

Bilateral limbal stem cell deficiency (LSCD) can be a rare manifestation in patients afflicted with xeroderma pigmentosum (XP). The authors report a rare case of a 12‐year‐old boy who presented with redness and defective vision and was diagnosed with bilateral LSCD and hyperpigmented lesion over the face and trunk suggestive of XP.

## 1 CASE DESCRIPTION

**FIGURE 1:**
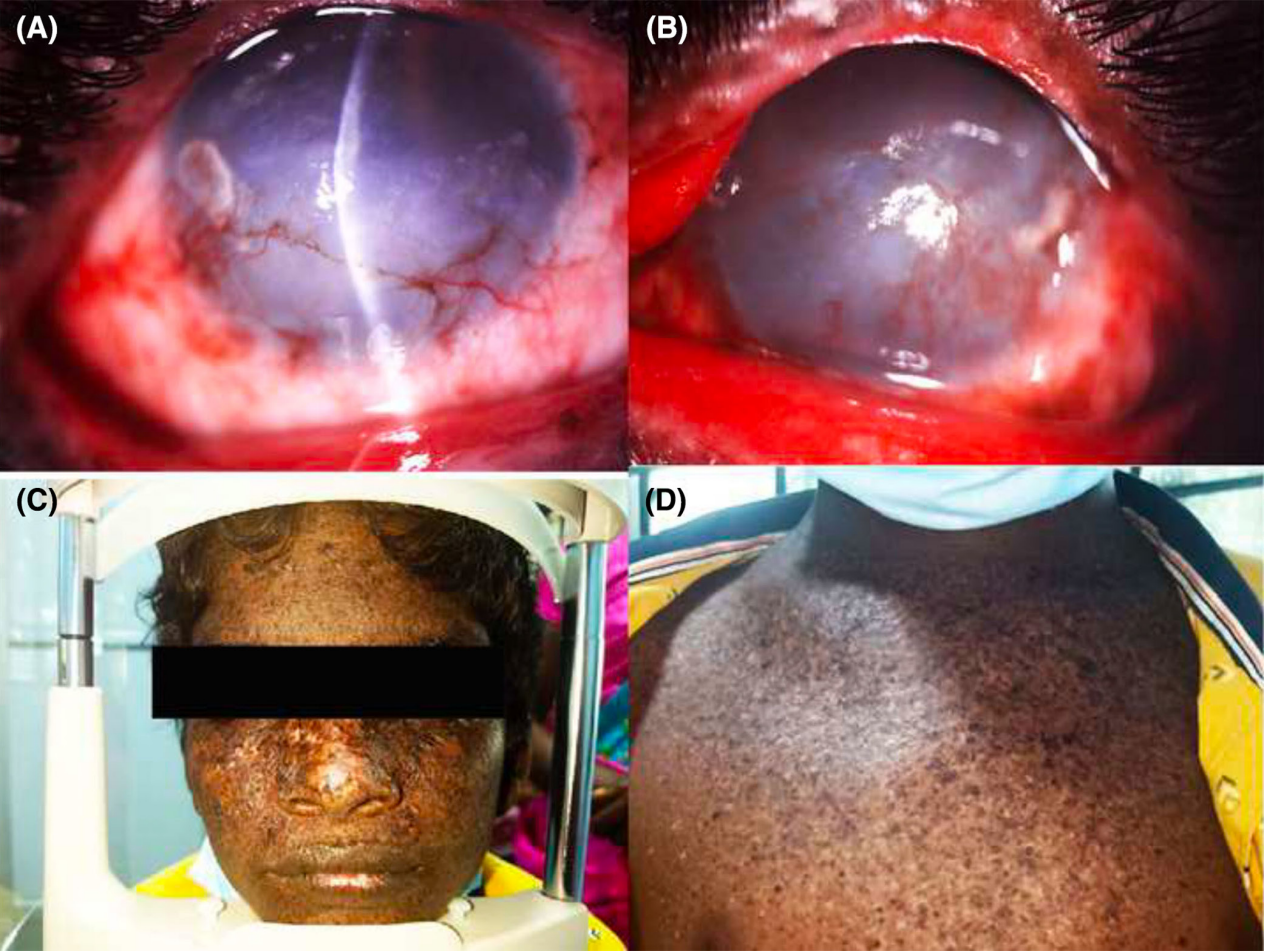
Digital slit lamp image of the right eye (A) and left eye (B) of the child depicting conjunctival hyperemia, congestion, superficial vascularization, stromal edema, patchy corneal scarring with 360‐degree limbal stem cell deficiency. Digital gross image of the child depicting diffuse hyperpigmented and depigmented patches over the face (C) and all over the trunk (D).

Xeroderma pigmentosum (XP) is a rare autosomal recessive genodermatosis with a deficiency of enzymes required for repairing UV‐induced DNA damage.^1^ In limbal stem cell deficiency (LSCD), there is a deficiency of limbal stem cells that maintain the corneal epithelium and provide a barrier function.^2^ A 12‐year‐old young boy presented with recurrent pain, redness, and defective vision in both eyes (OU) for the past 4 years. He gave a history of similar episodes in the past and was on treatment for pigmented lesions over his face and trunk since childhood. Anterior segment examination revealed conjunctival tarsal hyperemia, bulbar congestion, stromal edema, patchy scarring, superficial vascularization, and 360‐degree loss of Vogt palisades in OU (Figure 1A,B). Fundus was hazy and B scan was normal in OU. Systemic examination revealed hyper and depigmented lesions over the face and exposed areas of the trunk (Figure 1C,D). Corrected distant visual acuity was 20/1200 in the right eye and 20/200 in the left eye. He was diagnosed as XP with LSCD and was treated with topical 0.5% Moxifloxacin and 0.1% Dexamethasone combination in tapering doses, along with 1% Carboxymethylcellulose 4 times/day. Stromal edema improved in 2 weeks and vision returned to 20/120 in OU. He was planning for simple limbal epithelial transplantation along with optical penetrating keratoplasty in the future.

## AUTHOR CONTRIBUTIONS

**Bharat Gurnani:** Conceptualization; data curation; formal analysis; investigation; methodology; project administration; resources; software; supervision; validation; visualization; writing – original draft; writing – review and editing. **Kirandeep Kaur:** Conceptualization; data curation; formal analysis; investigation; methodology; project administration; resources; software; supervision; validation; visualization; writing – original draft; writing – review and editing.

## CONFLICT OF INTEREST STATEMENT

There are no conflicts of interest.

## CONSENT

Written informed consent was obtained from the patient to publish this report in accordance with the journal's patient consent policy.

## ETHICS STATEMENT

At our institute case reports, images and case series are exempted from IRB approval and the research followed the tenets of the Declaration of Helsinki.

## Data Availability

The patient details are available in the electronic medical records and can be made available from the authors on request.
